# Tom Connors

**DOI:** 10.1038/sj.bjc.6600292

**Published:** 2002-04-22

## Abstract

*British Journal of Cancer* (2002) **86**, 1205–1206. DOI: 10.1038/sj/bjc/6600292 www.bjcancer.com

© 2002 Cancer Research UK

## 

[Fig fig1]

**Figure fig1:**
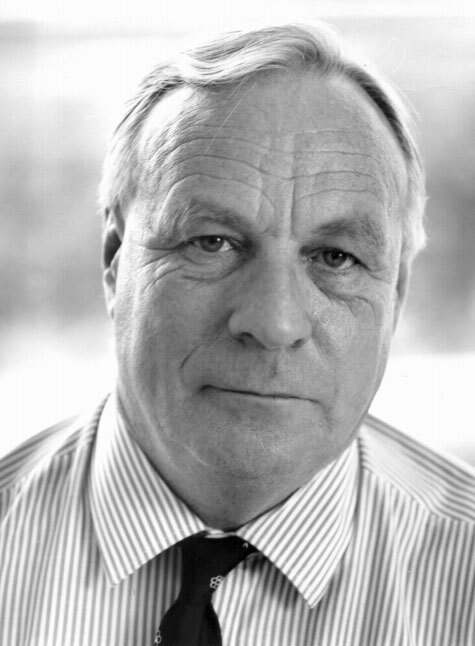
Thomas Anthony Connors BSc, PhD, DSc, FIBiol18 December 1934 – 4 February 2002

After a long battle with prostate cancer, Tom Connors died in his sleep, during the early hours of Monday 4th February: a true irony for someone whose research had contributed so much to the fight against cancer and who had made such an outstanding contribution to the whole cancer research community.

Tom was, without doubt, one of the best known and most popular figures in cancer research. He had been involved in the development of new anti-cancer drugs for 40 years and for the last 30 years he was one of the world's leading authorities. During his career he served on just about every National and International Committee of importance in his field and he had chaired most of them. He was the only non-American to ever serve on the President's advisory committee.

He was a member of the Editorial Board of the British Journal of Cancer for many years. Tom could be relied upon as a referee who would always give an honest and unbiased opinion.

Born in Mortlake, on the Thames west of London, in 1934, Tom was educated at Wimbledon College and University College London, graduating with a Special Physiology Honours degree in 1957. It was then that a family friend, one of the most eminent chemists ever to work in the field, the late Professor Walter Ross, persuaded him to consider cancer research as a career and to study with him at the Chester Beatty Research Institute. This Tom did, gaining his PhD in organic chemistry in 1960. So began his career in experimental cancer chemotherapy. Later his career would expand into toxicology, where he made a major contribution, gaining respect and admiration of the toxicology community. Between 1976 and 1991 he was the Director of the Medical Research Council's Toxicology Unit. Tom was a superb communicator and teacher; many of us have learned a great deal from him, at all stages of our careers. However, I do not think any of us have learned to be quite so bright and sparkling after a night drinking with him.

Always a great listener, Tom was receptive to new ideas and suggestions. But those of us who knew him well knew that his responses, whilst polite and constructive, were generally short and left no doubts of his opinion. A good idea would always be ‘brilliant’, anything less would be ‘interesting’. Many of today's senior cancer research workers passed through his hands, as students, post-doctoral fellows or visiting workers. His message was simple: without a sound understanding of the biology of the cancer process and the pharmacology of anti-cancer agents, it would be impossible to develop more effective anti-cancer therapies. His emphasis on this message within the scientific community was greatly appreciated by his clinical colleagues and has served to bring a unity of purpose to the whole process of developing new anti-cancer drugs.

It is difficult to single out specific achievements from the many in his scientific career. But his work with cis-platinum and its derivative carboplatin, two of the most effective anti-cancer agents, is recognised as outstanding. Tom Connors was the first person to evaluate the antitumour potential of cisplatin and his initiative led to its first clinical trials at the Royal Marsden Hospital. Later, in collaboration with the late John Roberts, he conducted fundamental studies into its mechanism of action. This in turn, led to the development of carboplatin, a derivative with good anti-cancer activity but with fewer unpleasant side effects.

During a long association with The Cancer Research Campaign, he served as Chairman of the Scientific Committee and the Grants Committee and several other important sub-committees. However, his most inspired role was in the formation of the CRC Phase I/II Drug Development Committee. The work of this committee has made Great Britain the world leader in the development of new anti-cancer drugs. Tom also served on the Committee of Management of the Institute of Cancer Research and on the Board of Governors of the Royal Marsden Hospital.

Tom will be remembered by many for his involvement in the British Association for Cancer Research (BACR). He attended nearly every BACR meeting after joining it in 1969. In 1990 he became Chairman of the BACR Executive Committee, a position that allowed him to play a major role in developing the Association into the internationally recognised body that it is today. He was instrumental in setting standards of excellence in the clinical and laboratory research presented at its annual meetings. His commitment to the BACR led to him being chosen as its President in 1994. Usually Presidents are merely figureheads, but Tom continued to involve himself in many aspects of the activities of the Association. His interaction with the younger members culminated in his co-producing the famous BACR Song-Book in 1995.

In 1997, to mark his services to the BACR and in recognition of his contribution to cancer research, the Executive Committee proposed that a special lecture should be presented at the Annual Meeting of the Association: ‘The Tom Connors Award Lecture’. This has now been presented four times by British speakers of international repute.

On his retirement, in 1994, Tom took up an appointment as Honorary Professor at the School of Pharmacy, University of London. From this position, and in his own time, he continued to make a major contribution to the development of new anti-cancer drugs. He served on many committees, made active contributions to scientific meetings and willingly gave his advice and guidance to others. Tom received many scientific and academic accolades, including honorary degrees from several universities and awards from learned societies. Sadly, he never received the wider public recognition that many felt he richly deserved.

His passing leaves a tremendous gap in the cancer research community. He will be sadly missed and warmly remembered by all the friends and colleagues who were privileged to know him as someone who was just ‘brilliant’.

*John Double*

